# Neural Oscillatory Dynamics in Joint Action: Dissociable Roles of Entrainment and Beta Modulation in Self–Other Integration

**DOI:** 10.1111/nyas.70314

**Published:** 2026-07-08

**Authors:** Mattia Rosso, Bavo Van Kerrebroeck, Peter Erik Keller, Marc Leman, Pieter‐Jan Maes, Peter Vuust

**Affiliations:** ^1^ Center for Music in the Brain, Department of Clinical Medicine Aarhus University & The Royal Academy of Music Aarhus/Aalborg Denmark; ^2^ IPEM – Institute for Psychoacoustics and Electronic Music Ghent University Ghent Belgium; ^3^ SPL – Sequence Production Lab McGill University Montreal Canada; ^4^ IDMIL – Input Devices and Music Interaction Laboratory McGill University Montreal Canada; ^5^ MARCS Institute for Brain, Behaviour and Development Western Sydney University Sydney Australia

**Keywords:** beta modulation, electroencephalography (EEG), hyperscanning, interpersonal synchronization, neural entrainment, oscillations, self–other integration

## Abstract

Temporal coordination is fundamental for human communication and collaboration, yet the underlying neural mechanisms remain poorly understood. Central to this process is self–other integration, defined here as the extent to which a partner is processed as self‐relevant and incorporated into one's sensorimotor representations. Recent evidence suggests that beta‐band oscillatory dynamics may provide a shared sensorimotor framework supporting such integration. Here, we leveraged an immersive virtual‐reality body‐swap illusion to experimentally manipulate the embodiment of a partner's hand during joint rhythmic action, thereby testing the sensitivity of oscillatory brain dynamics to distinct levels of self–other integration. Forty participants, paired into 20 dyads, performed a finger‐tapping task while viewing either their partner's hand in first‐person (1P) or second‐person (2P) perspective, or their own hand in uncoupled control conditions. Electroencephalography hyperscanning demonstrated that both neural entrainment of low‐frequency oscillations and beta modulation linked to partner‐generated movements occurred in visually coupled conditions. However, only beta modulation was selectively enhanced when participants perceived their partner's hand from a 1P perspective. These findings suggest that while neural entrainment reflects a general mechanism for tracking a partner's rhythmic behavior, beta modulation specifically supports the integration of the other's effector into one's bodily representation.

## Graphical abstract

We investigated how neural entrainment and beta power modulation contribute to interpersonal synchronization under different visuomotor contexts using a body‐swap paradigm. Neural entrainment tracked a partner's rhythmic behavior across conditions, whereas beta modulation was selectively enhanced when the partner's effector was perceived from a first‐person perspective. These findings indicate that complementary neural mechanisms support interpersonal coordination, with beta modulation specifically reflecting self–other integration at the sensorimotor level.

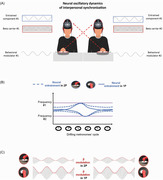

## 1 Introduction

The ability to put oneself in another's place is crucial for human cooperation and may be unique to our species [1]. This faculty, known in cognitive neuroscience as perspective‐taking [2], holds more than just a metaphorical meaning. Evidence from a range of task domains suggests that perspective‐taking is an embodied process, whereby the brain integrates information from the other in relation to one's own bodily state in order to infer their mental states and coordinate with their actions [3, 4, 5, 6, 7]. Understanding how the brain facilitates this process is one of the primary quests of contemporary social neuroscience [8].

Perspective‐taking can be experimentally induced and investigated with the body‐swap illusion [9, 10, 11]. In this paradigm, the visual perspective of two participants is switched via head‐mounted displays, such that one can perceive the other's body from the other's first‐person perspective (1P). This scenario differs from natural scenarios where individuals interact from a second‐person perspective (2P), namely, perceiving the other while maintaining one's own point of view [12]. This manipulation is particularly insightful because it is thought to trigger qualitatively different mechanisms of self–other integration [13], which is a critical component of human interactions [14, 15, 16, 17]. When the other's body is perceived in 1P, due to the spatiotemporal congruency between the visual percept (the other) and the afferent proprioceptive signals (the self), the other's body parts can be integrated into one's own mental body schema [11]. This process ties together perception and action relying on dynamic neural representations of the bodily self, based on sensorimotor recalibration [18, 19]. While visual manipulations have been extensively used to investigate how the embodiment of external limbs comes with illusory sense of ownership and agency (for reviews, see Refs [20, 21]), to our knowledge, no study to date has leveraged visual manipulations to uncover the neural dynamics underlying self–other integration and how these influence an ongoing social interaction.

Using the body‐swap illusion, Rosso et al. [13] showed that inducing mutual perspective‐taking between two individuals by switching their visual perspectives facilitates attraction toward a synchronized state, pointing to a dependency of temporal coordination on the integration of the other's body state into the individual sensorimotor systems. The authors proposed that assuming a forward model of the other's effector compels individuals to minimize the error between executed and observed action, which ultimately leads to an increase in behavioral synchronization [22]. The aim of the present study is to move beyond behavioral evidence and understand the neural mechanisms by which the motoric information produced by another individual is integrated into the sensorimotor system during interpersonal synchronization.

In order to address this question, we applied two recently developed electroencephalography (EEG) analysis methods [23, 24, 25, 26, 27] to a hyperscanning dataset collected in the joint finger‐tapping study of Rosso et al. [13]. The task was carried out in the “drifting metronomes” paradigm, where each member of the dyad was assigned a metronome and instructed to synchronize their finger taps with it. While the two metronomes were set to start in‐phase, a small difference in their tempo resulted in repeated cycles of dephasing. Despite the instruction to ignore the partner while maintaining synchronization with the assigned metronome, this setup has consistently resulted in unintentional synchronization among the partners across independent studies [13, 28, 29]. Furthermore, the continuous dephasing enabled the analysis of the time‐varying aspect of the neural dynamics as the interaction unfolds, through phases of coupling and decoupling characterizing metastable behavior [14, 30, 31, 32]. This experimental design allowed us to quantify the emergence of the neural dynamics of interest resulting from visual coupling and, importantly, to compare them across 1P and 2P modes of interaction.

Neural dynamics supporting joint action have been examined across multiple frequency bands, with interbrain synchrony commonly taken as an index of shared social cognition processes coactivating in the two brains. Notably, synchronization in the theta and low‐alpha range has been associated with the coordination of higher order cognitive processes during social interaction. Reports include theta/alpha (6–12 Hz) amplitude synchrony during alternating speech coordination in human communication [33], and theta‐band (4–7 Hz) phase synchronization related to the sense of joint agency during cooperative actions [34] and joint‐context statistical learning [35]. Dynamics in the alpha range have been linked to shifts in self–other attentional allocation and to coordinated versus uncoordinated interaction, indexed via both power modulation [36, 37, 38, 39] and interbrain synchronization [40, 41]. It should be noted that subsequent work did not consistently observe alpha‐range effects in interpersonal synchronization paradigms [24, 42], leading some authors to propose that alpha dynamics are unlikely to be generated within the motor systems of interacting individuals [42].

Since self–other integration is framed here as a fundamentally sensorimotor mechanism, we focused our analyses on two dominant endogenous rhythms co‐occurring in the sensorimotor system: low‐frequency delta (1–3 Hz) and beta (∼20 Hz) [43, 44, 45, 46]. Delta oscillations impose temporal constraints on the sampling of sensory information, dynamically aligning phase to facilitate the processing of rhythmic inputs and modulating behavioral outcomes [43, 47]. In an interpersonal context, the frequency range of delta rhythms is well matched to the natural rhythm of human motion, which may make observed biological movement a privileged class of rhythmic stimulus for the motor system in which to entrain [48, 49, 50]. Beta rhythms, in turn, are tightly linked to motor processing and have been associated with motor timing, prediction, internal action models, and the integration of observed movements with ongoing motor control. Notably, beta power modulation occurs both during self‐generated movements [51, 52] and in response to sensory input [53, 54, 55, 56], making it a candidate mechanism for regulating action timing in joint action [24, 42, 57, 58]. Operating at the interface of motor and sensory processes, beta modulation is thought to coordinate executed and perceived actions through a shared oscillatory mechanism [24, 59, 60, 61].

**FIGURE 1:**
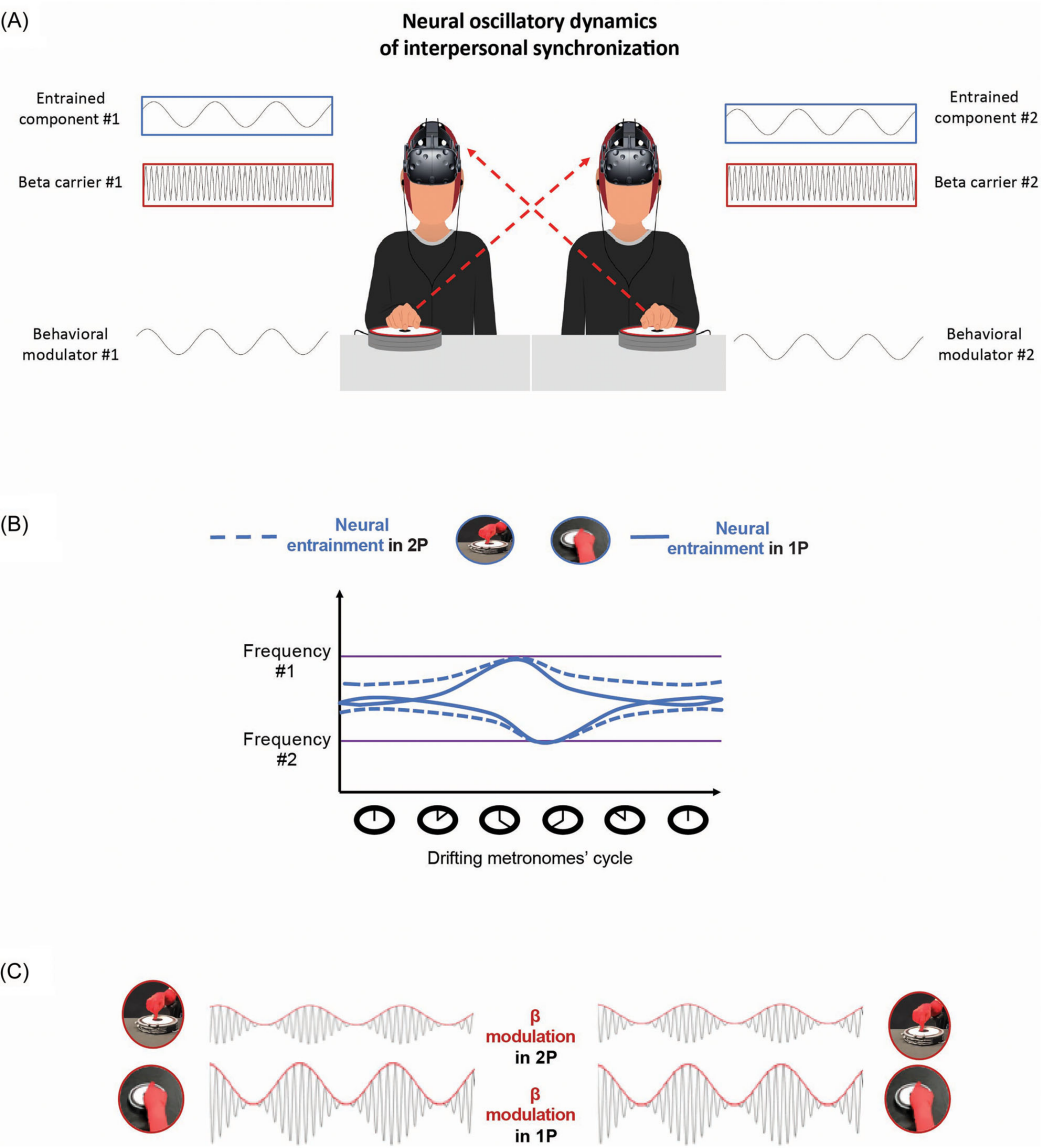
Schematic illustration of oscillatory neural dynamics underlying self–other integration during joint finger tapping. (A) *Oscillatory dynamics* under investigation are represented in the context of a joint finger‐tapping task, where participants are visually coupled by observing each other's tapping hand through a head‐mounted display. Participants observed their partner's hand movements either from a first‐person (1P) or a second‐person (2P) perspective while synchronizing with temporally incongruent auditory metronomes (*drifting metronomes*). For each participant in the dyad, we aimed to separate the following oscillations (from top to bottom): a low‐frequency component of the EEG signal attuned to the assigned metronome (entrained component), a high‐frequency component centered at 20 Hz (beta carrier), and a low‐frequency oscillation corresponding to the finger‐tapping cycles (modulator). The body‐swap manipulation affects how each participant processes the partner's movements as if they were their own (1P), allowing assessment of changes in the dynamics of interest compared to an ecological face‐to‐face interaction (2P). (B) *Neural entrainment* was quantified as frequency convergence between entrained components within dyads, across the drifting metronomes’ cycle. The two horizontal purple lines represent the ideal scenario in which each participant maintains their individual frequency based on the assigned metronome. The blue lines depict the expected alternating periods of frequency convergence and divergence across the cycle, modulated by visual coupling and perspective (dashed and solid lines represent 2P and 1P, respectively). (C) *Beta modulation* reflects periodic fluctuations in the power of ∼20 Hz oscillations driven by the partner's tapping. Modulation strength is expected to be higher in the 1P perspective, based on enhanced integration of the other's effector into the self's body schema as compared to an ecological 2P perspective. The curves are intended for illustrative purposes only.

A key novelty of the present work is that, for both frequency ranges, we move beyond hyperscanning approaches that rely on interbrain synchrony, which can remain opaque with respect to the mechanistic principles underlying observed cross‐brain correlations (for a recent critical review, see Ref [62]). Instead, we explicitly model frequency‐specific neural modulations as a function of sensory stimulation and partner behavior, thereby obtaining behavior‐referenced measures of low‐level sensorimotor integration during interpersonal synchronization and testing their sensitivity to visual coupling and embodied perspective‐taking. For each frequency band of interest, we operationalized a distinct mechanism based on the temporal features and expected interactions in our experimental setup (Figure 1).

The first candidate mechanism is *neural entrainment*, consisting of the phase alignment of low‐frequency brain oscillations, initially attuned to the assigned stimulation frequency, with the partner's behavior evolving at an incongruent frequency. Adhering to the precise definition of neural entrainment [25, 63, 64], we explicitly modeled the convergence of individual oscillatory components toward a common frequency within the dyad. Specifically, from each participant's EEG signal, we extracted components attuned to the assigned metronome frequencies (1.667 Hz or 1.641 Hz, depending on the assigned metronome) and computed the dynamic convergence of their instantaneous frequencies over time [25, 26, 27, 65]. To investigate these neural mechanisms, we manipulated Coupling (coupled and uncoupled) and Perspective (1P and 2P) in a within‐subjects design, allowing us to systematically assess how visual coupling and embodied perspective‐taking influence self–other integration.

Regarding the expected effects on neural entrainment, we hypothesized that *A1)* visual coupling would result in significantly stronger frequency convergence of the individual oscillatory components, that is, a closer convergence toward a shared frequency, facilitated by visual information originating from the partner [29, 66, 67, 68]. Furthermore, we expected that *A2)* periods of frequency convergence would alternate with periods of frequency divergence, reflecting the alternating periods of coupled and decoupled behavior previously reported in the drifting metronomes paradigm. Finally, we hypothesized that *A3)* frequency convergence would be stronger in 1P as compared to 2P, consistent with the enhanced behavioral synchronization reported by Rosso et al. [13] and reflecting greater self–other integration.

The second mechanism of interest is *beta modulation*, consisting of changes in the power of a ∼20 Hz oscillatory component in response to rhythmic events. Beta modulation was operationalized here as a form of brain‐to‐behavior coupling, quantified as the systematic variation of ∼20 Hz power as a function of the phase of the partner's finger taps. We hypothesized that *B1)* visual coupling would result in significant beta modulation driven by the partner's tapping cycles, and that *B2)* the modulation would be stronger in the 1P coupled condition as compared to the 2P coupled condition, reflecting enhanced integration of the partner's movements into one's own body schema due to embodied perspective‐taking.

**FIGURE 2:**
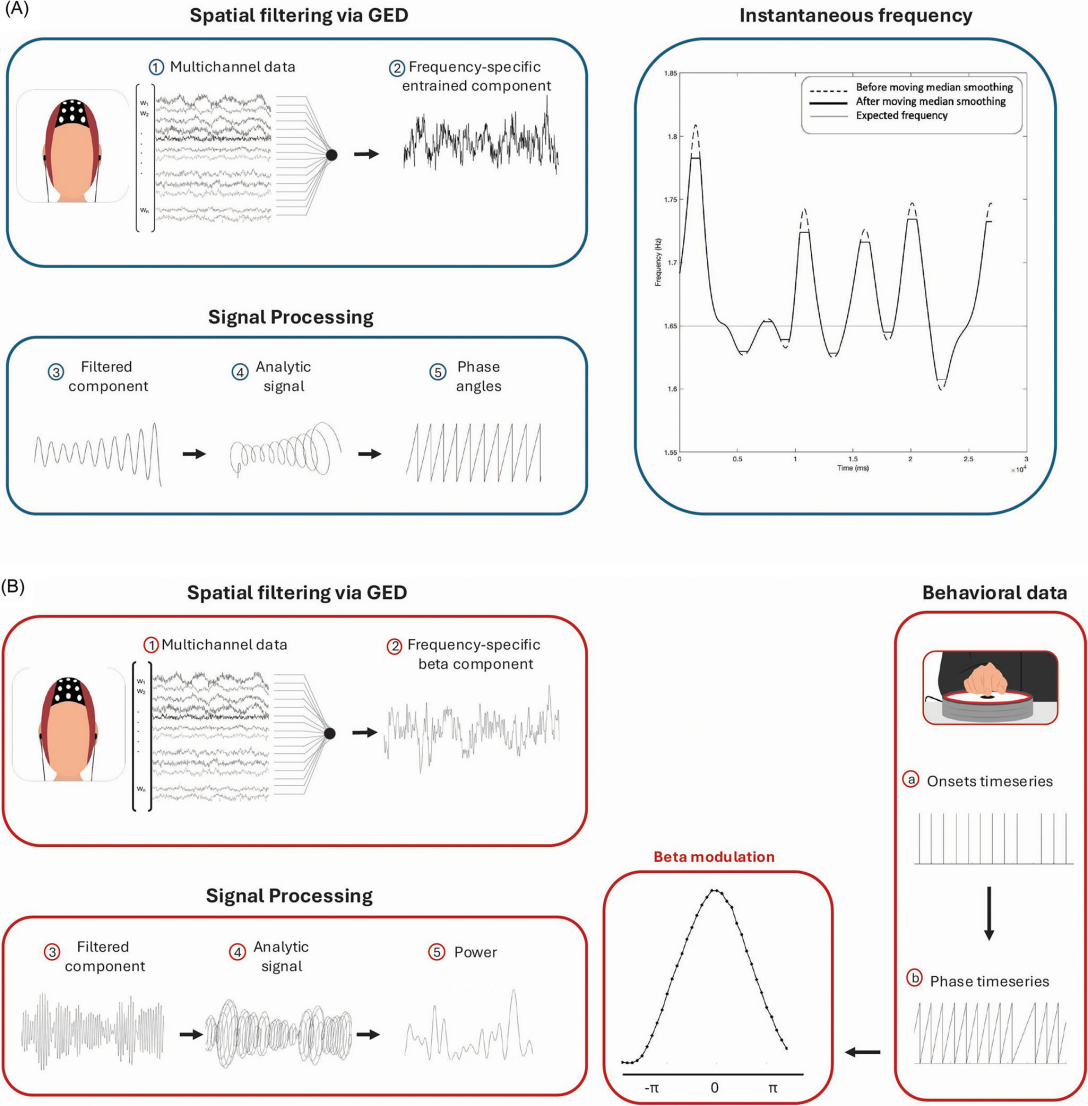
Signal processing pipelines for extracting oscillatory components from EEG hyperscanning data. (A) Neural entrainment pipeline. A spatial filter was computed using generalized eigendecomposition (GED) (1) to extract a frequency‐specific EEG component maximally attuned to the metronome's stimulation frequency (1.67 or 1.64 Hz, depending on partner), reducing the dimensionality to a single timeseries of interest. The resulting frequency‐specific component (2) was band‐pass filtered (3), and the analytic signal (4) was computed via Hilbert transform to extract instantaneous phase angles (5). Instantaneous frequency was derived from the temporal derivative of the phase timeseries and smoothed using a moving median filter. Applying this pipeline to each participant in the dyad allowed estimation of time‐resolved frequency fluctuations and their convergence across the metronomes’ cycles. (B) Beta modulation pipeline. A spatial filter was computed using GED (1) to extract an EEG component that best separates narrow‐band beta activity (∼20 Hz) from broad‐band activity. The resulting beta component (2) was band‐pass filtered (3), and the analytic signal (4) was computed via the Hilbert transform to extract beta power timeseries (5). In parallel, behavioral onset timeseries from the partner's finger taps (a) were converted into phase timeseries (b). Beta power was then binned as a function of tapping phase, and a sinusoidal function was fitted to quantify the strength of beta modulation driven by the partner's tapping cycles.

More generally, the present approach moves beyond traditional hyperscanning measures based on interbrain synchrony by coupling neural dynamics directly to observable behavior. By explicitly linking oscillatory activity to well‐defined behavioral events such as the partner's tapping cycles or the evolving phase of the drifting metronomes, it provides a more interpretable and mechanistic account of interpersonal interaction. Together, our hypotheses address both a general principle of error minimization between self and other [22] at the level of coupled neural dynamics through neural entrainment, and a specific form of sensorimotor integration via beta modulation. Support for these hypotheses would suggest that perspective‐taking enhances self–other integration by modulating multiple levels of neural processing. Figure 1 provides a visual representation of the dynamics under investigation and our hypotheses, while Figure 2 illustrates the analysis pipeline leading to their computation.

## 2 Methods

### 2.1 Participants

Forty (*N* = 40) right‐handed human participants, recruited through university mailing lists and local Facebook groups, took part in the study (28 females, 12 males; mean age = 31.42 years, standard deviation [SD] = 7.49 years). Individuals were divided into two gender‐matched groups and randomly paired in 20 dyads (*N* = 20) to control for gender bias in the interaction. Two dyads were excluded from the analysis: one because of failure to comply with the instructions, one because a participant requested to perform the behavioral task without undergoing EEG recording. None of the participants had a history of neurological, psychiatric, or major medical disorders, nor were professional musicians. All participants declared they did not know the assigned partner before the experiment took place. The study was approved by the Ethics Committee of Ghent University (Faculty of Arts and Philosophy) and informed written consent was obtained from each participant, who received a 20€ coupon as compensation for their participation.

### 2.2 Experimental Task

The behavioral task consisted of the drifting metronomes paradigm for dyadic entrainment, a joint finger‐tapping task described in detail in Refs [13, 28, 29]. The two partners in the dyad were seated at the same table, facing one another, and were instructed to tap their right index finger on a circular pad placed in front of them, in sync with an auditory metronome. Each partner was cued with a metronome set at a slightly different tempo (100 BPM and 98.5 BPM, or 1.667 Hz and 1.641 Hz), resulting in a pattern of linear dephasing. Specifically, the metronomes’ relative phase gradually increased from 0 to π radians, then decreased from π to 0 radians over the course of 10 consecutive cycles, for a total duration of 390 s. Participants were instructed to ignore any visual input and keep tapping along with the assigned metronome. During the task, participants were equipped with HTC Vive Pro 2 head‐mounted displays for immersive virtual‐reality environments, which we used to stream video recordings of their own (uncoupled conditions) or of the partner's hand (coupled conditions) in real time, from a first‐person (1P conditions) or second‐person (2P conditions) perspective. The combination of these factors resulted in the 2 × 2 experimental design (Coupling × Perspective). Experimental conditions were administered in two predefined blocks corresponding to visual perspective (2P and 1P), due to the need to reposition the camera when changing perspectives. Within each block, condition order was randomized, and block order was randomized across participants. See Ref [13] for more technical details of the system setup, validation of the visual perspective manipulation in VR, technical details of behavioral data capture and stimuli presentation, and additional data collected from the participants which are not reported in the present work.

### 2.3 Data Acquisition

Each participant was provided with a circular pad containing a strain gauge pressure sensor, used to detect tapping onsets with a 1‐ms resolution. For each dyad, two pads were connected to the same Teensy 3.2 microcontroller, which worked as a serial/MIDI hub to log contact times with the pad and communicate with the experimental PC and EEG system. Simultaneous EEG recordings were performed on both partners within each dyad during the entire experiment. At the beginning of each metronome's cycle, a TTL trigger was sent from the Teensy microcontroller to the EEG amplifiers via BNC connection, to synchronize behavioral and neural timeseries. Each participant was equipped with a 64‐channels waveguard TM original EEG headset (10–10 system, with Ag/AgCl electrodes). Two ANT‐Neuro eego TM mylab systems were connected with a trigger adapter cable provided by the manufacturer to synchronize the recordings. Recordings were performed at a sampling rate of 1 kHz. Each pair of headsets shared a common ground, while CPz_partner1 was used as common reference electrode for both partners. Impedances were monitored in the eego TM software environment and kept below 20 kΩ.

### 2.4 EEG Preprocessing

The EEG preprocessing pipeline was written integrating functions from the Fieldtrip toolbox [69] for MATLAB (MathWorks Inc, USA). For every dyad, data were acquired as a 128‐channels recording and split offline in 2 × 64‐channels subsets, individually re‐referenced to the electrodes CPz_partner1 and CPz_partner2, respectively. Following the rejection of bad channels, identified based on visual inspection of the raw timeseries and on the distribution of variance across channels, we re‐referenced the two recordings to the average of the respective 64 electrodes. This procedure prevented extreme noise from the removed bad channels to leak into the common average. On average, 4.9 bad channels (SD = 2.9) were removed per participant per experimental condition. A sixth‐order Butterworth high‐pass filter with 1 Hz cut‐off was applied to the raw recordings to remove slow drifts. This conservative threshold was taken given the long duration of the recordings and the motor task involved. As shown in Ref [25], these parameters of the high‐pass filter do not influence the oscillatory dynamics relevant to neural entrainment. A low‐pass sixth‐order Butterworth filter with 40 Hz cut‐off was applied to remove high‐frequency muscular activity. A fourth‐order notch filter centered at 50 Hz was applied to remove power‐line noise up to the third harmonic.

Subsequently, independent component analysis (ICA) [70, 71, 72] was conducted as implemented in the *runica* Fieldtrip algorithm. Stereotyped artifacts were identified by means of visual inspection of the components’ scalp topographies and activation timeseries. The reference CPz and the bad channels’ timeseries were excluded from the input data matrix. Under optimal conditions, removal would have been limited to the components exhibiting the stereotypical frontal distribution generated by blinks and lateral eye movements, or bilateral temporomastoidal distribution with periodic peaks in the activation timeseries attributable to heart beats. However, as compared to our previous report on the preprocessing of hyperscanning data in the same experimental paradigm [24], we opted for a more stringent approach and removed abnormal independent components characterized by sustained high‐frequency noise or recurrent transients in activation, and typically centrofrontal clusters. We point out that some of these artifactual components might be caused by interference of the setup with the HMDs and call for extra caution when cleaning EEG data collected under these conditions. However, we can safely conclude that eventual residual contributions of these artifacts were kept to a minimum, because centrofrontal clusters were excluded from the macro selection of regions of interest (ROI), while we reported significant differences across conditions which cannot be explained by residual noise. On average, 6.3 artifactual components (SD = 3.6) were identified and removed per participant per experimental condition. A visual comparison of the dataset before and after IC removal was carried out to assess the quality of the removal. Special attention was given to the frontal clusters of electrodes maximally contaminated by eye‐related artifacts. Rejected bad channels were reconstructed after artifact removal, by computing the average activity from neighboring electrodes indicated by the template provided by ANT‐Neuro for 64‐channel waveguard TM original caps. No segmentation in epochs was performed during this preprocessing phase, so that every experimental condition was treated as a continuous experimental run.

### 2.5 Generalized Eigendecomposition

Generalized eigendecomposition (GED) [73] was used to design a spatial filter in order to separate narrow‐band activity in the frequency ranges of interest from the background broad‐band activity [74], while reducing the dimensionality of the multivariate EEG dataset to one or a small set of component timeseries (details presented below). These extracted component activation timeseries (i.e., the estimation of the underlying source's activation) were used as input for the hyperscanning analyses presented in the next paragraph. Replicating the analysis pipelines adopted in our previous work, we extracted the entrained component maximally attuned to the stimulation frequency (1.667 Hz or 1.641 Hz, depending on the assigned metronome) and a narrow‐band beta component (20 Hz) from the individual EEG recordings. For the entrained components, the filter was designed in the frequency domain as a Gaussian function with central frequency of 1.667 Hz or 1.641 Hz (depending on the assigned metronome) and full width at half the maximum of 0.3 Hz [23, 25, 26, 27]. For the beta component, the filter was designed as a plateau‐shaped finite impulse response (FIR) filter centered at 20 Hz and covering a frequency range of 18–22 Hz (slope = 15%) [24]. For both components, the broad‐band EEG data were filtered via element‐wise multiplication between the spectrum of the broad‐band signal and the spectrum of the filter kernel, before transforming the resulting narrow‐band spectrum back into the time domain via inverse Fourier transform.

The EEG datasets were spatially filtered by weighting the channels timeseries. The set of vectors *W* (weights) was calculated by solving the following equation:

RWΛ=SW

where *S* is the covariance matrix of the narrow‐band signal; *R* is the reference covariance matrix of the broad‐band signal; Λ is the set of eigenvalues. GED identified the set of eigenvectors *W* that best separate the signal (“*S*”) covariance from the reference (“*R*”) covariance matrix. The *S* covariance matrix was computed from the narrow‐band signal, the *R* covariance matrix was here computed from the broad‐band multivariate signal.

The eigenvector (*w*) associated with the largest eigenvalue was taken as a spatial filter, transposed and multiplied by the broad‐band data matrix to reconstruct the activation timeseries (*y*) of our target components:

$$y=w^{T}\;X$$

The corresponding spatial projection of the components over the scalp (*a*) was computed as follows:

$$a=w^{T}\;S$$

Covariance matrices were computed within 600 ms time windows starting at the finger‐tap onsets, and grand‐average *S* and *R* covariance matrices were computed. Matrices whose z‐normalized Euclidean distance from the grand‐average exceeded the 2.23 z‐scores (corresponding to a probability of 0.01) were removed, and the grand‐average *S* and *R* were recalculated based on the remaining covariance matrices. To improve the numerical stability of the GED performance in case of rank deficiency, regularization was applied to the R covariance matrix by adding 1% of its average eigenvalues as a constant to its diagonal.

Besides optimizing the signal‐to‐noise ratio between narrow‐ and broad‐band neural activity, GED allows channel selection bias to be avoided. Aiming to replicate our previous work, the spatial filter was optimized based on a macroselection of ROIs, consisting of a large cluster of the 37 channels located behind the frontocentral “FC” line—mastoids excluded. The *S* and *R* covariance matrices were computed from this cluster of ROIs. The rationale was to provide full coverage of centroparietal, temporal, and occipital regions where the dynamics of interest were shown to be maximally expressed, while minimizing noise from less relevant sensors [23, 24].

To facilitate replication and reuse, we provide an open‐source implementation of the GED pipeline in the FREQ‐NESS toolbox [46], which supports both sensor‐level EEG and source‐reconstructed data.

### 2.6 Dyadic EEG Analyses

Following source separation performed on each participant, two distinct analyses were performed on the entrained (1.667 Hz or 1.641 Hz) and beta (20 Hz) components within each dyad, for each experimental condition. All neural outcomes reported here are within‐participant measures explicitly referenced to stimulation and behavioral signals, namely, the assigned metronome (for neural entrainment) or the partner's tapping cycles (for beta modulation). Although EEG was recorded simultaneously from both members of each dyad, we did not compute interbrain synchrony or coupling metrics (e.g., cross‐brain phase locking, coherence, or correlation). Because our hypotheses concern within‐brain, behavior‐referenced dynamics and our experimental design includes explicit uncoupled control conditions matched in sensory context and global temporal structure, we did not employ surrogate or shuffled‐pair procedures that are typically used to define null distributions for interbrain synchrony measures (see Ref [75]). In contrast with hyperscanning methods based on interbrain measures of synchrony [62], we advocate for explicitly modeling the dynamics of the ongoing interaction in order to provide mechanistically interpretable measures.

Neural entrainment within dyads was computed as the convergence of individual instantaneous frequency timeseries [23, 25, 26] toward a shared frequency. The same Gaussian filter used for GED was applied to the individual entrained components to extract reliable phase timeseries from the analytic signal [76], computed via Hilbert transform. In order to remove discontinuities caused by phase resets, the timeseries were unwrapped, differenced, and scaled to Hz [65]:

$$\;Hz_{t}=\frac{s\left(\phi_{t}-\;\phi_{t-1}\right)}{2\pi }$$

The resulting instantaneous frequency timeseries were smoothed with a sliding moving median (window width of 400 samples), to remove transient artifactual activity that may distort the phase timeseries [65].

For each dyad, a time‐varying measure of convergence was computed as the difference between the individual instantaneous frequency timeseries: 𝐻𝑧_𝑡__Sub#2 − 𝐻𝑧_𝑡__Sub#1. A value of 0 was expected in case of perfect convergence, whereas a value of −0.026 Hz was expected in case of maintenance of the assigned metronomes’ frequencies. The resulting convergence timeseries were segmented in 10 trials aligned with each full drifting metronomes’ cycle (0–2π) and divided into 64 consecutive phase bins. Individual samples were averaged within each bin. The resulting timeseries shared the same format as the behavioral measures analyzed in our previous work [13, 28, 29], allowing us to replicate the statistical analysis.

**FIGURE 3:**
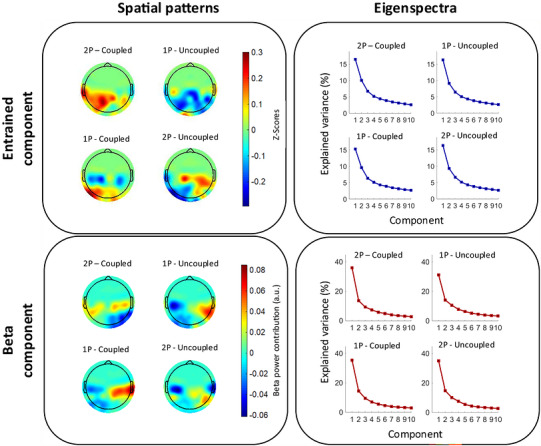
Generalized eigendecomposition (GED) assessment. For both the entrained component and the beta component, spatial activation patterns and eigenspectra are shown across the four experimental conditions (2P–coupled, 1P–uncoupled, 1P–coupled, 2P–uncoupled), averaged across all participants (*N* = 36, 18 dyads). Spatial activation patterns represent the topographical distribution over the scalp of the entrained (top) and beta (bottom) components. For the entrained component, spatial activation patterns were z‐score normalized across conditions before averaging, due to the presence of abnormal variance in some participants’ channels. Consistent with the manuscript's emphasis on temporal dynamics, we refrain from strong claims regarding the anatomical generators of the observed topographies. Nonetheless, activation over the right temporoparietal junction in the 1P coupled condition is noteworthy, as this region has previously been implicated in social interactions, particularly in low‐level processing such as the attentional allocation to salient stimuli and attribution of agency [82]. Eigenspectra show the proportion of explained variance for the first 10 components in each condition. For the entrained component, the leading GED eigenvalue clearly dominates, indicating successful separation of the target signal and justifying its use as a spatial filter. In contrast, the eigenspectra for beta components show the eigenvalues of the principal component analysis (PCA) applied to the top 10 GED beta components to extract the dominant source of variance in beta power. This additional step on beta components was motivated by the gradual decay of GED eigenvalues, suggesting that relevant dynamics were distributed across multiple components which we aggregated with PCA.

Beta modulation was computed for every participant based on the distribution of beta power as a function of the phase of the finger‐tapping cycles produced by the partner [24]. To compute beta power timeseries, the same plateau‐shaped FIR filter used for GED was applied to the beta components before performing the Hilbert transform. For each of the 37 beta components separated by GED, beta power was computed as the squared magnitude of the resulting analytic signal. Given the worse performance of GED for separating beta components as compared to low‐frequency entrained components [46], as indexed by the gradual decay of the eigenvalues in the beta range (see Figure S1), we applied principal component analysis (PCA) to the set of beta power timeseries extracted from the GED beta components to capture their shared variance in a low‐dimensional representation. The first principal component (PC1) was retained as a robust summary estimate of covarying beta power fluctuations across components, thereby minimizing information loss associated with variance being distributed across multiple GED components. PCA eigenspectra are reported in Figure 3. Extreme power values deviating from the mean by more than 3 SDs were considered outliers and removed from PC#1. Finger‐tapping phase timeseries were divided into 36 bins (bin size = 10°) [55], so that the median PC#1 values falling within the same bins were computed. With this procedure, we obtained the beta power curves as a function of the partner's movement cycles, computed over the total amount of finger‐taps (645 events were expected on average from each participant). Based on the observation that beta power modulation could be well approximated by a sinusoidal function across one full tapping cycle, the best‐fitting sinewave was estimated using the *sineFit* MATLAB function [77], for each participant in each experimental condition. The sine amplitude provided us with a measure of beta modulation strength, replicating the approach presented in [24].

Note that neural entrainment and beta modulation are indexed using different phase references, and therefore different segmentation schemes. Neural entrainment is segmented relative to the drifting metronomes’ phase cycle and discretized into 64 bins to yield a time‐resolved convergence trajectory aligned to metronome relative phase. This choice matches the format of our time‐resolved behavioral coupling measures, thereby enabling direct brain–behavior correlations [13, 28, 29]. In contrast, beta modulation is computed relative to the partner's tapping‐cycle phase (0–2π) across taps within each condition run (645 events expected on average per participant). For this event‐based analysis, we adopted 36 bins (10° resolution), consistent with prior work quantifying beta modulation as a function of bodily effectors [24, 51, 52, 55], thereby facilitating replicability. The processing pipelines for computing neural entrainment and beta modulation are illustrated in Figure 2.

### 2.7 Statistical Modeling

For neural entrainment, we used the frequency difference timeseries as the response variable in a mixed‐effects model. The model included Coupling and Perspective as factors, and Time as a continuous predictor expressed as the indexes of the metronome steps (from 1 to 64 in one drifting metronomes’ cycle). Due to the nonlinearity of the curves, we employed the method of orthogonal polynomials [78], incorporating linear and quadratic functions of Time into our model (second‐order polynomial). Random effects were specified to capture heterogeneity in entrainment trajectories over Time, aiming for a maximal structure to minimize false‐alarm rates without substantial loss of power [79, 80]. However, because adding dyad‐level random effects in addition to the Dyad × Condition grouping term yielded singular fits, we report the maximal nonsingular random‐effects structure supported by the data, with random intercepts and Time trends varying by Dyad × Coupling × Perspective. The full model's formula was defined as follows:

$$\begin{array}{ccc} & & \mathrm{Fre}q_{\mathrm{diff}}\;∼\;\left(\mathrm{Time}\;+\;\mathrm{Tim}e^{2}\right)\;\times \;\mathrm{Coupling}\;\times \;\mathrm{Perspective}\;\\ & & +\;(\mathrm{Time}\;+\;\mathrm{Tim}e^{2}\;|\;\mathrm{Dyad}:\mathrm{Coupling}:\mathrm{Perspective}) \end{array}$$

For beta modulation, the sine amplitudes quantifying beta modulation strength were log‐transformed to ensure residuals met the assumptions of normality (*p* = 0.057, Shapiro–Wilk test).

$$\mathrm{Bet}a_{\mathrm{Modulation}}∼\;\mathrm{Coupling}\;\times \;\mathrm{Perspective}\;+\;(1\;|\;\mathrm{Subject}:\mathrm{Dyad})$$

Factors were leveled such that uncoupled and 2P would provide the baseline intercept for the model, since the modulation by the other's movements was expected to be null in absence of coupling between the partners. The interaction between individual Subjects and the respective Dyad was modeled as a random effect.

To assess the association between neural dynamics and behavioral synchronization, we modeled behavioral synchronization as a function of neural entrainment and beta modulation using linear mixed‐effects models. Behavioral synchronization was quantified with recurrence score, a time‐varying measure of dyadic coordination aligned with the drifting metronomes cycle (details in Refs [13, 28, 29]). Neural entrainment entered the models as a time‐resolved predictor. Beta modulation was first estimated separately for each participant and condition, then averaged across the two members of each dyad within each condition to obtain a dyad‐level beta estimate. This dyad‐level beta estimate was log‐transformed and assigned to all corresponding time points. All continuous variables were *z*‐scored prior to model fitting. Models included dyad as a random intercept to account for nonindependence of observations within dyads.

To test whether the relationship between neural dynamics and behavioral synchronization varied as a function of Coupling and Perspective, we fitted three nested models. First, we specified a baseline model including only task structure:

$$\mathrm{Recurrenc}e_{\;t}∼\mathrm{Coupling}\;\times \;\mathrm{Perspective}+\left(1|\mathrm{Dyad}\right)$$

Second, we fitted a model including neural predictors as condition‐invariant main effects:

$$\begin{array}{ccc} & & \mathrm{Recurrenc}e_{\;t}∼\;\mathrm{FreqDif}f_{\;t}+\;\mathrm{Beta}\;+\;\mathrm{Coupling}\;\times \;\mathrm{Perspective}\;\\ & & +\left(1|\mathrm{Dyad}\right) \end{array}$$

Third, to test whether neural–behavior associations differed across experimental conditions, we fitted a full interaction model:

$$\begin{array}{ccc} & & \mathrm{Recurrenc}e_{\;t}∼\;(\mathrm{FreqDif}f_{\;t}+\mathrm{Beta})\;\times \;\mathrm{Coupling}\;\times \;\mathrm{Perspective}\;\\ & & +\left(1|\mathrm{Dyad}\right) \end{array}$$

Model comparisons were performed using likelihood‐ratio tests. This allowed us to assess, first, whether neural predictors explained behavioral variance beyond task structure, and second, whether allowing the slopes of neural entrainment and beta modulation to vary as a function of Coupling and Perspective improved model fit. Model‐level explained variance was quantified using marginal *R* ^2^.

EEG processing and analyses were entirely carried out in MATLAB (version R2019a). Statistical analyses were carried out in R (version 4.5.0), using the *lme4* package [81] for model fitting.

## 3 Results

We found that both neural entrainment and beta modulation were elicited when partners in dyads performed the finger‐tapping task in visually coupled conditions. However, each mechanism responded selectively to our manipulation of visual perspective, suggesting they play distinct roles in the integration of motoric information produced by the partner during interpersonal synchronization.

### 3.1 Neural Entrainment

**FIGURE 4:**
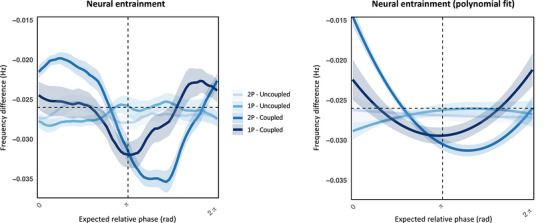
Neural entrainment as frequency convergence. The plot on the left shows neural entrainment quantified as the difference in instantaneous frequency (Hz; *y*‐axis) between the entrained components of each partner's EEG signal, computed over the drifting metronomes’ cycle (*x*‐axis, expressed as expected relative phase in radians from 0 to 2π). Continuous lines indicate the average frequency difference computed from all drifting metronomes’ cycles and from all participants (*N* = 36); shaded areas indicate the standard error of the mean (SEM) across participants. Values closer to 0 indicate stronger convergence between partners’ neural signals. Across all visually coupled conditions, oscillatory components converged toward a shared frequency around the in‐phase attractor point and diverged after the antiphase midpoint. As expected, both 2P and 1P uncoupled conditions fluctuated around −0.026 Hz, which is the expected difference based on the difference in the metronomes’ frequencies. Instead, both 2P and 1P coupled conditions showed a clear parabolic pattern of convergence, with a significant quadratic modulation. Against our predictions, the overall depth of convergence remained unaffected by perspective, as quantified by the nonsignificant interaction between the quadratic term of the model, Coupling, and Perspective. However, 2P coupling exhibited more asymmetric convergence–divergence slopes, while the 1P perspective rebalanced this asymmetry, leading to more symmetric convergence dynamics. The plot on the right represents the quadratic polynomial fit to the same curves, highlighting the asymmetry in convergence across 1P and 2P coupled conditions. Group‐level generalized eigendecomposition spatial maps and eigenspectra for the entrained component are shown in Figure 3.

Neural entrainment was quantified as the convergence of instantaneous frequencies between the two partners’ entrained EEG components over time. The entrained component was robustly isolated by GED, with consistent group‐level spatial maps and a dominant leading eigenvalue across conditions (see Figure 3). We found a significant two‐way interaction effect between Coupling and the quadratic term of Time (Estimate = 0.025, SE = 0.006, *p* < 0.001), indicating that frequency convergence in coupled conditions was modulated as a function of the drifting metronomes’ cycle. As shown in Figure 4, the oscillations maximally converged toward a shared frequency around the in‐phase attractor point and diverged toward the respective metronome's frequencies after the antiphase point. This parabolic modulation enabled us to model the time‐varying component of convergence using polynomial fitting [78] (for more details on the statistical model, see Section 2). Furthermore, we found a significant two‐way interaction between Coupling and the linear term of Time, capturing the symmetry between the rates of divergence and convergence (Estimate = −0.025, SE = 0.007, *p* < 0.001). As shown in Figure 4, frequencies reached maximum divergence after the antiphase midpoint in the coupled 2P condition, followed by a steeper attraction as they re‐entered in phase at the other end of the cycle. This pattern closely resembles the asymmetric behavioral coordination dynamics consistently reported in independent studies using the drifting metronomes paradigm [13, 28, 29]. Specifically, the in‐phase attractor exerted a longer lasting influence on neural convergence as participants attempted to dephase away from its region.

Against our initial hypothesis, we did not find any significant interaction effect between Perspective and the quadratic component of the modulation, indicating that the depth of frequency convergence was invariant between 1P and 2P coupling. However, the significant three‐way interaction between Coupling, Perspective, and the linear term of Time (Estimate = 0.023, SE = 0.010, *p* = 0.019) shows that visual perspective influenced the asymmetry of the dynamic described above. When participants were coupled in 1P, the rates of divergence from and convergence toward the in‐phase point became significantly more symmetric. Table 1 shows the fixed‐effects parameter estimates, their standard errors, and associated *p* values. Uncoupled (factor Coupling) and 2P (factor Perspective) were taken as 0‐levels for statistical contrasts.

**TABLE 1:** Neural entrainment results.

Term	Estimate	Std. error	*t* value	*p* value	Significance
(Intercept)	−0.027	0.000	−50.524	0.000	***
ot1	−0.002	0.005	−0.445	0.656	
ot2	0.001	0.004	0.138	0.890	
Perspective	0.000	0.001	0.061	0.951	
Coupling	−0.000	0.001	−0.201	0.841	
ot1:Perspective	0.007	0.007	1.059	0.290	
ot2:Perspective	−0.004	0.006	−0.760	0.447	
ot1:Coupling	−0.025	0.007	−3.666	0.000	***
ot2:Coupling	0.025	0.006	4.147	0.000	***
Perspective:Coupling	0.000	0.001	0.110	0.912	
ot1:Perspective:Coupling	0.023	0.010	2.353	0.019	*
ot2:Perspective:Coupling	−0.002	0.008	−0.235	0.814	

Results from a linear mixed‐effects model examining neural entrainment as a function of drifting metronomes’ cycles (ot1 and ot2, polynomial terms of Time), visual perspective (1P vs. 2P), and movement coupling (coupled vs. uncoupled). The model was fitted to the frequency difference over the drifting metronomes’ cycle, and includes fixed main effects and interactions. Significant interaction effects between factors and polynomial terms indicate modulation of neural phase alignment by sensorimotor perspective and interpersonal coupling. Significance thresholds: *p < 0.05, ***p < 0.001.

### 3.2 Beta Modulation

**FIGURE 5:**
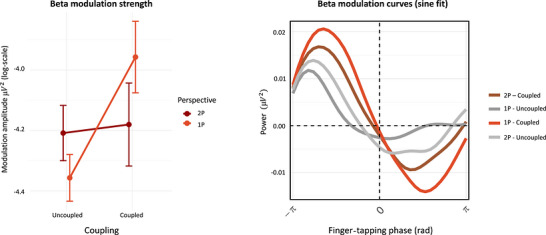
Beta modulation as a function of the partner's movement cycles. The plot on the left shows the average beta modulation strength computed across 36 participants (*N* = 36) in each experimental condition. Mean amplitude of beta‐band modulation (log‐transformed; *y*‐axis) is shown in response to the partner's tapping cycle, plotted across levels of Coupling and color‐coded by Perspective (2P vs. 1P). Beta modulation was quantified by fitting a sine function to the time‐varying beta power aligned to the partner's tapping phase. A significant interaction was observed between Coupling and Perspective, driven by a larger increase in beta modulation in the 1P coupled condition compared to 2P. This suggests enhanced beta dynamics when participants viewed the partner's movements from a first‐person perspective while visually coupled. Error bars represent standard errors of the mean (SEM). The plot on the right shows the corresponding group‐averaged beta modulation curves, aligned to the partner's tapping phase (*x*‐axis, in radians), with a fitted sine wave for each condition. The curves reveal the temporal structure of beta power modulation, with peaks and troughs in alignment with the partner's movement cycle. The 1P coupled condition exhibits the clearest oscillatory profile, consistent with increased modulation strength, while the other conditions show reduced modulation patterns. Group‐level generalized eigendecomposition spatial maps and eigenspectra for the beta component are shown in Figure 3.

Beta modulation was quantified as the amplitude of the beta power distribution over the partner's finger‐tapping cycles. Group‐level GED and PCA quality metrics for the beta component, including spatial maps and eigenspectra, are reported in Figure 3. PC1 explained 34.39% of beta power variance on average across participants and conditions (see full eigenspectra in Figure 3 and Supporting Information). We found a significant two‐way interaction between Coupling and Perspective (Estimate = 0.371, SE = 0.178, *p* = 0.037), indicating that coupling with the partner in 1P resulted in significantly stronger beta modulation as compared to 2P. The interaction corresponded to a moderate standardized effect (model‐based Cohen's *d* = 0.69), with wide confidence intervals (95% CI [0.02, 1.36]), indicating substantial variability across dyads. This effect extends the findings reported by Rosso et al. [24], showing that a larger allocation of neural resources is devoted to tracking the partner's effector when it is perceived from a perspective that facilitates integration into one's own body schema. The results and interactions in the model are represented in Figure 5. Table 2 reports the fixed‐effects parameter estimates from the beta modulation model. Uncoupled (factor Coupling) and 2P (factor Perspective) were taken as 0‐levels for statistical contrasts.

**TABLE 2:** Beta modulation results.

Term	Estimate	Std. error	*t* value	*p* value	Significance
(Intercept)	−4.209	0.107	−39.333	0.000	***
CouplingCoupled	0.028	0.127	0.223	0.824	
Perspective1P	−0.149	0.127	−1.172	0.242	
CouplingCoupled:Perspective1P	0.372	0.179	2.077	0.038	*

Results from a linear mixed‐effects model predicting the strength of beta‐band modulation (∼20 Hz) by behavioral tapping phase. The model includes fixed effects of visual perspective, movement coupling, and their interaction. Significant beta modulation in the coupled × 1P condition suggests enhanced integration of the partner's tapping cycles into the self's motor representation when visual perspective is first‐person and sensorimotor coupling is present. Significance thresholds: *p < 0.05, ***p < 0.001.

### 3.3 Brain–Behavior Associations

Alongside our analyses focused on neural measures, we examined whether neural entrainment and beta modulation were associated with behavioral coordination, and whether these associations varied as a function of task structure. A baseline model including only Coupling, Perspective, and their interaction already accounted for a substantial proportion of behavioral variance (marginal *R* ^2^ = 0.393; conditional *R* ^2^ = 0.502), confirming that behavioral synchronization was strongly shaped by the experimental manipulation. In this model, Coupling showed a strong positive effect (Estimate = 1.210, SE = 0.029, *p* < 0.001), while the Coupling × Perspective interaction was significant (Estimate = 0.087, SE = 0.042, *p* = 0.037), whereas Perspective alone was not (Estimate = −0.012, SE = 0.029, *p* = 0.671). Thus, behavioral synchronization was strongly enhanced by visual coupling, with a modest additional advantage for the 1P compared with the 2P coupled condition. Adding neural entrainment and beta modulation as condition‐invariant predictors significantly improved model fit relative to the task‐only model (likelihood‐ratio test: χ^2^(2) = 849.17, *p* < 0.001), increasing explained variance to marginal *R* ^2^ = 0.493 (conditional *R* ^2^ = 0.585). In this model, both neural entrainment (Estimate = 0.313, SE = 0.010, *p* < 0.001) and beta modulation (Estimate = 0.042, SE = 0.014, *p* = 0.003) showed overall positive associations with behavioral synchronization.

Critically, however, allowing these neural–behavior associations to vary as a function of Coupling and Perspective yielded a further substantial improvement in fit (likelihood‐ratio test: χ^2^(6) = 514.4, *p* < 0.001), resulting in the best‐fitting model (marginal *R* ^2^ = 0.540; conditional *R* ^2^ = 0.630). This indicates that the predictive relationship between neural dynamics and behavioral synchronization was not constant across task conditions. In the full interaction model, neural entrainment remained a robust positive predictor of behavioral synchronization (Estimate = 0.128, SE = 0.029, *p* < 0.001), but its association with behavior was significantly modulated by task structure, as indicated by interactions with Coupling (Estimate = 0.403, SE = 0.033, *p* < 0.001), Perspective (Estimate = −0.146, SE = 0.042, *p* < 0.001), and their three‐way interaction (Estimate = −0.179, SE = 0.046, *p* < 0.001). Specifically, the entrainment–behavior relationship was strongest in the coupled 2P condition, remained positive in the coupled 1P condition, and was markedly weaker in uncoupled trials. Thus, higher neural entrainment values, reflecting greater convergence of the neural frequency‐gap measure, were most strongly associated with behavioral coordination when participants were visually coupled.

Beta modulation showed a distinct and more context‐dependent pattern. Although its global main effect was not significant in the full interaction model (Estimate = 0.027, SE = 0.029, *p* = 0.360), its association with behavioral synchronization significantly depended on task condition, as indicated by a significant interaction with Coupling (Estimate = 0.075, SE = 0.031, *p* = 0.015) and a significant three‐way interaction with Coupling and Perspective (Estimate = −0.222, SE = 0.048, *p* < 0.001), while the interaction with Perspective alone was not significant (Estimate = −0.021, SE = 0.040, *p* = 0.607). Notably, beta modulation showed a positive association with behavioral synchronization in the coupled 2P condition, but a negative association in the coupled 1P condition and near‐zero associations in uncoupled conditions. This pattern indicates that beta modulation is not uniformly related to overt coordination but instead tracks behavioral synchronization in a condition‐specific manner, with its relationship to behavior reversing depending on visuomotor context. Together, these findings indicate that neural entrainment provides a consistent and robust marker of behavioral synchronization, particularly under visual coupling, whereas the behavioral relevance of beta modulation is more conditional and context‐dependent. These results are reported in Table 3.

**TABLE 3:** Brain–behavior associations as a function of the experimental design structure.

Predictor	Estimate	Std. error	*t* value	*p* value	Significance
(Intercept)	−0.613	0.073	−8.358	< 0.001	***
Neural entrainment	0.128	0.029	4.356	< 0.001	***
Beta modulation	0.027	0.029	0.916	0.360	
Coupling	1.139	0.026	44.576	< 0.001	***
Perspective	−0.018	0.029	−0.619	0.536	
Coupling × Neural entrainment	0.403	0.033	12.239	< 0.001	***
Coupling × Beta modulation	0.075	0.031	2.431	0.015	*
Perspective × Neural entrainment	−0.146	0.042	−3.505	< 0.001	***
Perspective × Beta modulation	−0.021	0.040	−0.513	0.607	
Coupling × Perspective	0.235	0.041	5.793	< 0.001	***
Coupling × Perspective × Neural entrainment	−0.179	0.046	−3.862	< 0.001	***
Coupling × Perspective × Beta modulation	−0.222	0.048	−4.654	< 0.001	***
Model‐level marginal *R* ^2^					0.540

Linear mixed‐effects model predicting behavioral recurrence score from neural entrainment and beta modulation, including main effects and interactions with Coupling and Perspective. The model includes a random intercept for dyad. Model‐level explained variance is reported as marginal *R* ^2^. Significance thresholds: *p < 0.05, ***p < 0.001.

To summarize, neural entrainment manifested as the frequency convergence of individual oscillatory components initially attuned to the assigned metronomes as a result of visual coupling. Across the drifting metronomes cycle, this dynamic was susceptible to the influence of attractor points, alternating between phases of convergence and divergence. Convergence strength was invariant to visual perspective, while the convergence and divergence slopes were more similar in 1P coupling. Beta modulation strength, on the other hand, increased significantly when participants were coupled in 1P compared to 2P. Brain–behavior associations revealed a dissociation between the two neural mechanisms. Neural entrainment showed a consistent positive association with behavioral synchronization, whereas the relationship between beta modulation and behavior was strongly context‐dependent, reversing between 2P and 1P coupling. The differential sensitivity of neural entrainment and beta modulation to visual perspective, together with their distinct relationships to behavior, supports a functional specialization of the two mechanisms underlying interpersonal synchronization.

## 4 Discussion

With the present work, we showed that distinct oscillatory neural dynamics underpin distinct functions in interpersonal synchronization, integrating different dimensions of the motoric information produced by a human partner. While both neural entrainment and beta modulation were elicited in all conditions of visual coupling, beta modulation was selectively enhanced when participants perceived their partner's hand from a first‐person perspective (1P), as if it belonged to their own body. Our results suggest that neural entrainment and beta modulation contribute to interpersonal synchronization in functionally distinct ways. While neural entrainment appears to provide a robust mechanism for tracking and behaviorally aligning to a partner's rhythmic actions, beta modulation appears to index a more specific sensorimotor integration process linked to how the partner's effector is incorporated into one's bodily representation.

By examining these mechanisms under the body‐swap illusion [9, 10], we have shown that beta modulation, in particular, is enhanced under conditions that induce a recalibration of the sensorimotor system, such as perceiving the partner's hand from a first‐person perspective (1P; see Figure 5). This enhanced modulation suggests that beta rhythms are not merely a generalized mechanism for tracking rhythmic stimuli [53, 54, 83, 84] and perceived actions [24, 55, 57, 58, 61]. Instead, they might play a crucial role in dynamically integrating spatiotemporal information about the current body state to update one's body schema [18, 19, 85]. The alignment of visual and proprioceptive cues within the 1P condition creates the minimal and sufficient conditions needed for incorporating an external body part into a coherent sense of self‐location and body ownership [86, 87, 88]. Importantly, this integration is strengthened by visuomotor synchronization, namely, when the perceived effector moves in congruence with the observer's motor commands and sensory predictions [89, 90, 91]. We propose that beta oscillations facilitate this sensorimotor integration by being rhythmically modulated as a function of the partner's movement cycles.

Beta modulation has been shown to differentiate contributions by the self and the other to joint action within the motor system based on different degrees of beta power suppression [61], and has been proposed as a shared oscillatory mechanism for pacing one's own movements as well as tracking those of others [24]. It is noteworthy that the increment in modulation in 1P suggests that such differentiation no longer holds in a condition where the partner's actions are integrated into the self's body‐schema. The concept of self–other integration is paramount to interpersonal coordination, as integrating motoric information from another person's body into the perceiver's motor program enables individuals to adjust their actions in response to the other's movements [14, 15, 22, 39, 92]. As a prominent endogenous rhythm in the motor cortex and supplementary motor area [44, 46], beta oscillations are well‐positioned to support this integration due to their role in sustaining motor execution and control through corticomuscular coupling [93]. Beta rhythms uniquely coordinate both efferent motor commands along the corticospinal pathway and afferent signals that inform the brain about the body's current state, enabling continuous recalibration and stabilization [94, 95, 96, 97]. This dual function of beta oscillations, encoding outgoing and tracking incoming signals, makes them ideally suited to handle the dynamic requirements of incorporating another's movements into one's own motoric framework.

Novembre et al. [42] previously reported that inducing 20 Hz interbrain synchrony between two individuals’ motor cortices aligned sensorimotor processing within dyads. The alignment of excitability phases of beta oscillations between the two brains influenced the timing of joint action initiation and improved the likelihood of achieving interpersonal movement synchrony. This finding suggests that beta oscillations play a causal role in coordinating motor actions between interacting individuals [98]. While the authors specifically focused on phase‐alignment, they put forward that the envelope of a 20 Hz carrier modulated by a movement frequency, as we computed in our study, would account for the contextual timing of the dyadic interaction [42]. According to this view, simultaneous movement would be promoted by alternating windows of motor excitability and inhibition coupled to the partner's tapping [99, 100]. In line with this evidence, we propose that sensorimotor integration aligns efferent motor commands to the timing of the other's effector, thereby shaping how self‐ and other‐generated action signals are coordinated during interaction. Importantly, our brain–behavior analysis indicates that this process is not uniformly expressed as better overt synchronization across all conditions. Instead, beta modulation appears to track condition‐specific sensorimotor processes whose behavioral consequences depend on visuomotor context. While inducing a bottom‐up embodiment of the partner's effector in 1P is sufficient to elicit stronger beta modulation at the individual level, this increase does not translate into a uniformly positive relationship with dyadic behavioral synchronization. The behavioral relevance of beta modulation appears to be strongest in more ecological 2P modes of interaction, whereas in 1P it may reflect sensorimotor integration processes that are not directly expressed in increased overt coordination. Notably, this more ecological 2P interaction mode closely matches the visuomotor configurations employed in previous studies supporting a causal role of beta‐band dynamics in interpersonal coordination, suggesting that beta modulation is selectively recruited for behaviorally effective coordination in such ecological contexts.

Although hyperscanning studies typically rely on interbrain synchrony as a neural index of dyadic alignment, it is important to clarify how we view this index relative to the measures used in our work, and why we opted for more readily interpretable alternatives. While interbrain synchrony in a frequency band of interest can be informative, it is inherently correlational and often remains mechanistically underdetermined. Notably, it does not by itself specify the behavioral driver or the directionality of coupling, which are explicitly modeled in our analyses (see Figure 1). As argued by Novembre and colleagues, interbrain synchrony can become functionally interpretable when it is experimentally induced (e.g., via dual‐brain stimulation) and thereby constrained and linked to a specific mechanism [42, 98]. In the absence of such causal manipulation, we leveraged the features of our experimental paradigm to index how one participant's sensorimotor activity is modulated as a function of the partner's effector dynamics, yielding a transparent and behaviorally grounded account of self–other integration.

Unlike beta modulation, which appears to be more tightly linked to self–other integration at the sensorimotor level, our findings indicate that neural entrainment supports synchronization with external stimuli without necessarily incorporating them into the self's motor schema. This distinction was highlighted by the body‐swap illusion, where the frequency convergence of entrained EEG components remains unaffected, suggesting that neural entrainment operates independently from sensorimotor recalibration. Instead, it tracks the partner's effector as a distal stimulus rather than integrating it into the self‐schema. By aligning internal oscillatory dynamics to the other's rhythmic movement, neural entrainment can support sensorimotor synchronization [25] without necessarily entailing changes in body representation.

This dissociation can be framed in relation to computational accounts of sensorimotor synchronization that distinguish multiple, interacting control mechanisms rather than a single hierarchical route to coordination [57]. For instance, in the ADaptation and Anticipation Model (ADAM), reactive error correction is implemented in an adaptation module that adjusts timing via period and phase correction, whereas predictive processes are implemented in an anticipation module that generates temporal estimates of upcoming events [101, 102, 122]. The output of the adaptation module informs an internal model of the self, which plays a role in action planning, while the output of the anticipation module feeds into an internal model representing the other that predicts a partner's future action timing. Critically, ADAM further proposes a joint internal model that compares the outputs of these internal models of self (action planning) and other (event prediction), and applies anticipatory error correction to reduce discrepancies by adjusting one's own plan prior to execution. Such operation is explicitly discussed as reflecting the relative balance of self–other integration and segregation [103]. Viewed through this lens, our results do not suggest that neural entrainment and beta modulation are competing explanations. Rather, they are consistent with the idea that interpersonal coordination engages complementary mechanisms operating at different levels: (i) a low‐frequency alignment to the partner's rhythmic behavior that tracks external temporal structure and is consistently associated with overt coordination, and (ii) a sensorimotor process that is selectively strengthened when the partner's effector is treated as self‐relevant, plausibly reflecting stronger coupling between action planning and predictions about the other as envisioned by joint‐model accounts of coordination. Notably, our brain–behavior results suggest that the behavioral expression of this latter process is context‐dependent rather than uniformly facilitative. Under maximal self–other integration in 1P, stronger beta modulation may reflect increased sensorimotor integration of the partner's effector which does not necessarily translate into tighter overt synchronization between individuals. By contrast, in more ecological 2P interaction contexts, beta modulation may be more selectively recruited to support interpersonal coordination itself.

In our study, the entrained oscillations under analysis fall within the delta range (0.5–4 Hz), an intrinsic rhythm prominent in the motor system alongside beta rhythms [43, 44], and well matched to the natural rhythm of human motion [48]. Since observing another person moving naturally overlaps with the perceiver's endogenous delta rhythms in motor areas, the proximity in frequencies renders human rhythmic motion a unique class of social rhythmic stimulus, which provides a natural affordance for the motor system to entrain [49]. To set the initial conditions for individual brains to converge toward a shared frequency, our drifting metronomes paradigm introduced a frequency gap between participants’ assigned metronomes. By extending recent advances in multivariate EEG analysis [23, 25] to hyperscanning recordings, we operationalized neural entrainment in the interpersonal domain following its fundamental definition [63, 76, 104]. Specifically, we separated each participant's oscillatory component most attuned to their metronome and tracked its instantaneous frequency changes throughout the task, to quantify convergence and divergence dynamics as the drifting metronomes’ cycle unfolded.

As shown in Figure 4, in both 2P and 1P visually coupled conditions, these oscillatory neural components diverged from the assigned metronome's frequency, gravitating instead toward the partner's frequency as the dyad approached the in‐phase attractor in the drifting metronomes’ cycle. Around the antiphase midpoint, partners decoupled, realigning their oscillatory component with their own metronome. This cyclical pattern of convergence and divergence, shaped by attractor points, mirrors the same attractor landscape underlying behavioral synchronization [13, 28, 29], suggesting that neural entrainment is a core mechanism in this process. While the overall extent of frequency convergence was consistent across visual perspectives, the balance between convergence and divergence rates varied. Specifically, coupling in 2P conditions produced a slower divergence followed by a steeper convergence, whereas 1P conditions balanced these dynamics, eliminating the directional effect seen in 2P (or hysteresis [105]). This nuanced dynamic does not alter our conclusions on the extent of the convergence, but it suggests subtle differences based on visual perspective that could be further explored through computational modeling of dwelling times, hysteresis, and memory effects in the system, to better clarify the underlying mechanisms. Importantly, a similar rebalancing was empirically observed at the behavioral level [13], reinforcing the close link between neural entrainment dynamics and behavioral synchronization, which was further confirmed by the present brain–behavior analysis.

Consistent with prior findings [43, 106, 107] and with the dynamic attending theory [108, 109], we propose that cortical delta dynamics [47, 110, 111], particularly their phase [112, 113, 114, 115], regulate attentional focus on competing rhythmic sensory inputs, namely, the assigned metronome versus the partner's movements. Translated to the interpersonal domain, participants appear to track each other's movements and guide their own behavior toward synchronized states through frequency convergence. This framework implies that dynamic allocation of attentional resources to competing inputs mediates transitions between cooperation and competition processes [28, 30], otherwise described as metastable behavior within the dyad [14, 31]. While our experiment cannot establish causality between oscillatory brain mechanisms and individual or dyadic behavior, the motor origin of delta oscillations has led authors to argue that active behavioral engagement drives cortical phase alignment, thereby enhancing sensory prediction [43, 110, 116]. In line with this argument, our results suggest that neural entrainment may facilitate prediction error minimization by reducing the temporal mismatch between executed and observed actions [22], consistent with its robust positive association with behavior across task contexts.

Together, our findings highlight that distinct neural modulation mechanisms integrate distinct action components perceived from another human in order to support interpersonal synchronization. These mechanisms are frequency modulation for delta (i.e., neural entrainment) and power modulation for beta (i.e., beta modulation) rhythms. Each frequency range plays a specific role in mediating temporal coordination between individuals, with delta oscillations tracking general rhythmic alignment and showing a robust relationship with overt behavioral synchronization, and beta oscillations selectively recalibrating the sensorimotor representation of the other's effector in a way whose behavioral relevance depends on task context. This distinction underscores how synchronization between humans relies on qualitatively different neural processes working together to achieve interpersonal coordination.

### 4.1 Limitations and Future Directions

Despite the novel contributions of this study, some limitations must be acknowledged. First, our ability to infer the precise brain networks underlying the observed oscillatory dynamics is constrained by the limited spatial resolution of EEG. Consequently, our discussion focused on temporal dynamics without speculating on the spatial extent of the neural networks involved. Future studies should take this limitation into account and apply different data acquisition methods and analytical approaches to overcome it. We point out that the recent development of network estimation via source separation (NESS) provides a promising path forward to integrate the temporal and spatial levels of analysis, by separating the sources of oscillatory activity in anatomical source space. Now adapted for frequency‐resolved (FREQ‐NESS [46, 117, 118, 119]) and broadband (BROAD‐NESS [120, 121, 123]) analysis of whole‐brain voxel data reconstructed from magnetoencephalography, this framework could enable future studies to capture the spatiotemporal characteristics of brain networks engaged in self–other integration during social interactions.

In addition, an alternative interpretation of our beta modulation findings relates to the perception of sensorimotor mismatches. Rather than reflecting the integration of an external effector per se, enhanced beta modulation in the 1P condition could stem from increased sensitivity to mismatches between action and perception, which are expected to be strongest in 1P visual coupling. These mismatches may disrupt phase‐aligned synchronous activity or alter phase‐resetting dynamics in neural populations. While our experimental design focused on dyadic interaction, future studies could test this alternative hypothesis in single‐participant setups, by introducing dynamic delays in the visual feedback of one's own movements to isolate the influence of perceived sensorimotor discrepancies from the integration of external body parts.

Importantly, while our experimental manipulations effectively elicited the oscillatory neural dynamics of interest, the findings remain correlational and therefore do not allow strong conclusions regarding their causal influence on dyadic behavior. Establishing causal links between specific neural mechanisms and behavioral outcomes will require intervention‐based approaches such as neurostimulation [42, 98] or computational modeling.

Finally, from an applied perspective, our results show that technology‐mediated manipulations (e.g., the body‐swap illusion) can selectively modulate candidate neural processes implicated in self–other integration. These observations motivate future work to test whether and how such targeted modulation translates into measurable changes in interpersonal coordination and functional outcomes in applied contexts such as physical therapy, neurorehabilitation, sports, and music training.

## 5 Conclusion

Our work builds on a line of behavioral research in interpersonal synchronization, which has shown that interactions can be shaped by manipulating the flow of information between individuals [13, 28, 29, 66, 67, 68]. By extending recent developments in multivariate EEG analysis [24, 25, 26, 46, 73] to a hyperscanning context, we delineated the distinct functional roles of neural entrainment and beta modulation in interpersonal synchronization.

To conclude, we underscore that rhythmic social interactions naturally induce diverse forms of neuromodulation by engaging mechanisms such as neural entrainment and beta modulation. These findings contribute to understanding the neural mechanisms that support interpersonal coordination and highlight how they can be modulated by changes in perceptual coupling.

## Author Contributions

Mattia Rosso conceived the hypotheses and designed the study. Mattia Rosso and Bavo Van Kerrebroeck conceived and implemented the experimental setup. Mattia Rosso and Bavo Van Kerrebroeck collected the data. Mattia Rosso performed preprocessing and data analysis. Mattia Rosso and Marc Leman performed statistical analysis. Peter Erik Keller, Marc Leman, Pieter‐Jan Maes, and Peter Vuust provided essential help to interpret and frame the results within the neuroscientific literature. Mattia Rosso and Peter Vuust wrote the first draft of the manuscript. Mattia Rosso prepared the figures. All the authors contributed to and approved the final version of the manuscript.

## Conflicts of Interest

The authors declare no conflicts of interest.

## Supporting information




**Figures S1 and S2**: nyas70314‐sup‐0001‐FigureS1‐S2.docx

## Data Availability

The preprocessed data that support the findings of this study are openly available on Zenodo: https://zenodo.org/records/20492602. All analysis scripts are publicly available at: https://github.com/mattiaRosso92/Oscillatory_Dynamics_Joint_Action. The GED implementation used here is also available in the FREQNESS toolbox, at: https://github.com/mattiaRosso92/Frequency‐resolved_brain_network_estimation_via_source_separation_FREQ‐NESS/tree/main/FREQNESS_Toolbox.
