# Supplementary material for: Neural Oscillatory Dynamics in Joint Action: Dissociable Roles of Entrainment and Beta Modulation in Self–Other Integration
Source: Ann N Y Acad Sci. 2026 Jul 8;1561(1):e70314. doi: 10.1111/nyas.70314 (PMC13344869; doi:10.1111/nyas.70314)
Supplement: Supplementary file 1 — Figures S1 and S2: nyas70314‐sup‐0001‐FigureS1‐S2.docx [file NYAS-1561-0-s001.docx]

***Supplementary Figure 1. GED eigenspectra for beta-band components.*** *Group-averaged eigenspectra obtained from generalized eigendecomposition (GED) applied to beta-band activity (18–22 Hz), shown separately for each experimental condition (2P–Coupled, 1P–Uncoupled, 1P–Coupled, 2P–Uncoupled). Plotted values indicate the proportion of variance explained by the first 10 GED components. Across all conditions, eigenvalues exhibit a gradual decay rather than a single dominant component, indicating that beta-band activity relevant to the task is distributed across multiple spatial components. This contrasts with the low-frequency entrained component, for which a clearly dominant leading eigenvalue was observed (see Figure 3 in the main manuscript). The absence of a sharply dominant beta component motivated the subsequent aggregation of beta power time series across GED components using principal component analysis (PCA), in order to capture shared, co-varying beta-power fluctuations while minimizing noise.*

 ***Supplementary Figure 2. Spatial activation patterns of the leading beta principal components.*** *Topographical distributions of the first nine principal components (PC#2–PC#10) obtained from principal component analysis (PCA) applied to beta-band power time series extracted from GED beta components. For each PC, scalp activation patterns are shown separately for the four experimental conditions (2P–Coupled, 1P–Uncoupled, 1P–Coupled, 2P–Uncoupled). Across components, spatial activation coefficients progressively decrease with component rank, mirroring the exponential decay observed in the PCA eigenspectrum. This pattern indicates that the leading principal component captures the dominant source of shared beta-power variance across GED components, while higher-order PCs contribute increasingly marginal variance. Consistent with the manuscript’s emphasis on temporal dynamics and power modulation, these spatial patterns are reported for completeness and quality assessment, without strong anatomical interpretation.*
